# Involvement of ceramide biosynthesis in increased extracellular vesicle release in *Pkd1* knock out cells

**DOI:** 10.3389/fendo.2022.1005639

**Published:** 2022-10-10

**Authors:** Valentina Carotti, Jenny van der Wijst, Eric H. J. Verschuren, Luco Rutten, Nico Sommerdijk, Charlotte Kaffa, Vera Sommers, Juan P. Rigalli, Joost G. J. Hoenderop

**Affiliations:** ^1^ Department of Physiology, Radboud Institute for Molecular Life Sciences, Radboud University Medical Center, Nijmegen, Netherlands; ^2^ Department of Biochemistry, Radboud Institute for Molecular Life Sciences, Radboud University Medical Center, Nijmegen, Netherlands; ^3^ Electron Microscopy Center, Radboud Institute of Molecular Life Sciences, Radboud University Medical Center, Nijmegen, Netherlands; ^4^ Radboud Technology Center for Bioinformatics, Radboud Institute of Molecular Life Sciences, Radboud University Medical Center, Nijmegen, Netherlands; ^5^ Department of Clinical Pharmacology and Pharmacoepidemiology, Heidelberg University Hospital, Heidelberg, Germany

**Keywords:** Autosomal Dominant Polycistic Kidney Disease, ADPKD, exosomes, extracellular vesicles, purinergic signaling, extracellular ATP

## Abstract

Autosomal Dominant Polycystic Kidney Disease (ADPKD) is an inherited disorder characterized by the development of renal cysts, which frequently leads to renal failure. Hypertension and other cardiovascular symptoms contribute to the high morbidity and mortality of the disease. ADPKD is caused by mutations in the *PKD1* gene or, less frequently, in the *PKD2* gene. The disease onset and progression are highly variable between patients, whereby the underlying mechanisms are not fully elucidated. Recently, a role of extracellular vesicles (EVs) in the progression of ADPKD has been postulated. However, the mechanisms stimulating EV release in ADPKD have not been addressed and the participation of the distal nephron segments is still uninvestigated. Here, we studied the effect of *Pkd1* deficiency on EV release in wild type and *Pkd1^-/-^* mDCT15 and mIMCD3 cells as models of the distal convoluted tubule (DCT) and inner medullary collecting duct (IMCD), respectively. By using nanoparticle tracking analysis, we observed a significant increase in EV release in *Pkd1^-/-^* mDCT15 and mIMCD3 cells, with respect to the wild type cells. The molecular mechanisms leading to the changes in EV release were further investigated in mDCT15 cells through RNA sequencing and qPCR studies. Specifically, we assessed the relevance of purinergic signaling and ceramide biosynthesis enzymes. *Pkd1^-/-^* mDCT15 cells showed a clear upregulation of *P2rx7* expression compared to wild type cells. Depletion of extracellular ATP by apyrase (ecto-nucleotidase) inhibited EV release only in wild type cells, suggesting an exacerbated signaling of the extracellular ATP/P2X7 pathway in *Pkd1^-/-^* cells. In addition, we identified a significant up-regulation of the ceramide biosynthesis enzymes *CerS6* and *Smpd3* in *Pkd1^-/-^* cells. Altogether, our findings suggest the involvement of the DCT in the EV-mediated ADPKD progression and points to the induction of ceramide biosynthesis as an underlying molecular mechanism. Further studies should be performed to investigate whether *CerS6* and *Smpd3* can be used as biomarkers of ADPKD onset, progression or severity.

## Introduction

Autosomal Dominant Polycystic Kidney Disease (ADPKD) is a multisystemic disorder characterized by the progressive bilateral development of renal cysts, which ultimately leads to end-stage renal disease (1, 2). The main cause of ADPKD are mutations in the *PKD1* gene, which encodes the protein polycystin-1 (PC1). ADPKD represents the main genetic cause of kidney failure worldwide (3). Hypertension and the consequent development of cardiovascular abnormalities contribute significantly to ADPKD morbidity and mortality (4). The onset of hypertension, mainly due to the activation of the renin-angiotensin-aldosterone system takes place in ADPKD patients even before the development of kidney symptoms (4–6).

Besides the presence of mutations in the *PKD1* gene or, eventually, in the *PKD2* gene, the onset and progression of ADPKD is associated with the activation of multiple cellular pathways that ultimately affect cellular proliferation and apoptosis, fluid secretion, inflammation and ciliary function. Several studies demonstrated the involvement of extracellular ATP and purinergic signaling in cyst development, as reviewed recently (7, 8). Extracellular ATP is released by the cyst-lining epithelium and accumulates in the cystic fluid (9–11), where it activates two groups of purinergic receptors, the ionotropic P2X receptors and the G-protein-coupled P2Y receptors (12). The expression of purinergic receptors (as for example P2X7) ﻿ (13) as well as ectonucleotidases such as CD39 is dysregulated in the context of ADPKD (14). P2X7 contribution to cyst development has been observed in different mouse models of PKD (15, 16). Similar findings were described in zebrafish (17, 18). However, the exact molecular and, especially, cellular mechanisms leading to the development of renal cysts and their focal nature are still not fully elucidated. Moreover, the age of onset of ADPKD can strongly differ between patients (19). So far, no biomarkers able to predict ADPKD onset and progression are available.

Extracellular vesicles (EVs) represent a heterogenous family of lipid vesicles carrying several cellular components (e.g., lipids, nucleic acids, proteins), which play an essential role in cell-to-cell communication. In the kidney, EVs have been shown to mediate proximal-to-distal communication and, in this way, regulate electrolyte transport (20). EVs comprise microvesicles and exosomes, which arise from the outward budding of the plasma membrane as well as from the endosomal compartment, respectively (21). The modulation of the EV release by purinergic signaling has been observed in several cell and animal models. Particularly, the activation of P2X7 leads to an increase in EV biogenesis, as demonstrated, for example, in macrophages (22), microglia (23), astrocytes (24) and cancer cells (25). So far, the association between purinergic signaling and EV release in the kidney has been minimally investigated. Furthermore, EV release is shown to depend on intracellular ceramide levels (26). Ceramide species can be generated *de novo* in a process catalyzed by ceramide synthase enzymes such as CERS6 as well as *via* the hydrolysis of the membrane lipid sphingomyelin, a reaction catalyzed by sphingomyelinases (18).

The disruption of the *Pkd1* gene has been associated with an increase in the EV release in mouse inner medullary collecting duct cells (mIMCD3) (27). In addition, Ding et al. described an increase in urinary EVs in ADPKD patients and studied the role of EVs in the development of the cystic phenotype *in vitro* and *in vivo* (28). Nevertheless, the molecular mechanisms leading to this increase in the EV release is not fully understood. To further elucidate the role of EVs in ADPKD development in the distal segments of the nephron, we evaluated the EV release in wild type and *Pkd1* knockout *(Pkd1^-/-^)* mouse distal convoluted tubule (mDCT15) and mIMCD3 cells, as well in an *in vivo* murine model of ADPKD. Furthermore, we investigated the effect of *Pkd1* deficiency in mDCT15 cells in relation to EV release, with a focus on the possible role of extracellular ATP and purinergic signaling as well as the modulation of ceramide biosynthesis in *Pkd1^-/-^* cells.

## Materials and methods

### Cell culture

mDCT15 cells were a kind gift from Dr. Robert Hoover (Emory University, Atlanta, USA) (29). The *Pkd1* ^-/-^ mDCT15 cells were generated by CrisprCas9 gene editing as previously described (30). The mIMCD3 cells (wild type and *Pkd1* ^-/-^) were a kind gift from Dr. Dorien Peters (Leiden University Medical Center, Leiden, the Netherlands) (31).

Wild type and *Pkd1* ^-/-^ mDCT15 cells were cultured in Dulbecco’s Modified Eagle Medium/Nutrient Mixture F12 Ham (DMEM/F12; Life Technologies, Carlsbad, USA) supplemented with 5% (v/v) fetal bovine serum (FBS) and 1% (v/v) penicillin-streptomycin. Wild type and *Pkd1* ^-/-^ mIMCD3 cells were cultured in DMEM-F12 (Sigma Aldrich, Burlington, USA) supplemented with 10% (v/v) FBS, 1 mM sodium pyruvate and 1% (v/v) penicillin-streptomycin. All cell lines were cultured at 37 °C in an incubator with 5% (v/v) CO_2_ and humidified atmosphere, trypsinized and passaged every 3-4 days.

### EV isolation from cell culture by ultracentrifugation

EVs from the culture medium of wild type and *Pkd1^-/-^* mDCT15 and mIMCD3 cells were isolated by ultracentrifugation as described previously (20, 32). Briefly, cells were plated in 8 T-175 flasks (1.2x10^6^ cells/flask for mDCT15 cells and 1.4x10^6^ cells/flask for mIMCD3 cells). After 24 hours, cells were washed with phosphate buffered saline (PBS) and cultured for further 24 hours in FBS-depleted medium (20 mL/flask). Afterwards, the medium (i.e., conditioned medium) was collected and centrifuged 15 minutes at 300 g. The supernatant was further centrifuged 30 minutes at 2,000 g. The resulting supernatant was then ultracentrifuged for 45 minutes at 12,000 g and filtered with a Ministart® 0.22 μm syringe filter (Sartorius, Göttingen, Germany). Finally, EVs were pelleted by ultracentrifugation at 108,000 g for 75 minutes, resuspended in EV-free PBS and centrifuged again at 108,000 g for 65 minutes. The EVs were resuspended in 100 µl of EV-free PBS. All ultracentrifugation steps were performed using a Sorvall WX+ Ultra Series ultracentrifuge (Waltham, Massachusetts, USA) and a swinging-bucket rotor AH629-36 (ThermoFisher Scientific, Waltham, Massachusetts, USA). All centrifugation and ultracentrifugation steps were performed at 4 °C. EV suspensions from 6 different batches were pooled, aliquoted and stored at -80 °C. EVs were characterized by nanoparticle tracking analysis and cryo-electron microscopy (see below).

### EV isolation from cell culture by precipitation

To confirm the effect of the knockout of *Pkd1* on EV release, a second isolation method based on a different physicochemical principle was used. Briefly, cells were plated in 6-well plates (1.5x10^5^ cells/well for mDCT15 cells and 1.75x10^5^ cells/well for mIMCD3 cells) and cultured for 24 hours in complete medium. Subsequently, cells were washed with PBS and further cultured in FBS-depleted medium for 24 hours. The medium was collected and centrifuged at 2,000 g for 30 minutes. The collected supernatant was mixed with the Total Exosome Isolation Reagent (from cell culture media) (Invitrogen, Waltham, USA) in 2:1 ratio (supernatant:reagent) and incubated overnight at 4 °C. EVs were pelleted by centrifugation (10,000 g, 1 hour) and resuspended in 100 µl of EV-free PBS. EV suspensions were stored at -80 °C.

### Extracellular ATP measurements

Wild type and *Pkd1* ^-/-^ mDCT15 cells were treated with apyrase (6 U/ml) or diluent (PBS) during the last 24 hours of culture. Extracellular ATP levels in the culture medium of control and apyrase-treated cells were quantified by chemiluminescence using the ATP Lite kit (Perkin-Elmer, Waltham, MA, USA), following manufacturer’s instructions. Additionally, EVs were isolated as described in the previous section.

### EV isolation from urine

To elucidate the effect of the loss of *Pkd1* expression on EV release *in vivo*, 24 hours-urine samples of wild type and iKsp-*Pkd1^-/-^* mice were used from a previous study from our group (33). Urinary EVs were isolated using the Total Exosome Isolation Reagent (from urine) (Invitrogen, Waltham, USA) following the manufacturer’s instructions.

### Gene expression analysis

Total RNA was obtained using the RNeasy Mini Kit (Qiagen, Hilden, Germany) according to the manufacturer’s instructions and stored at -80 °C. cDNA was generated from RNA samples using Moloney Murine Leukemia Virus Reverse Transcriptase (Thermo Fisher Scientific, Waltham, USA) according to the manufacturer’s instructions. cDNA samples were stored at -20 °C until further use. Gene expression was analyzed by Real-time qPCR, using the iQ SYBR Green Supermix (Bio‐Rad Laboratories, Hercules, CA, USA) on a CFX96 detection system (Bio‐Rad Laboratories, Hercules, CA, USA). The thermal profile consisted of an initial incubation of 3 minutes at 95 °C, followed by 40 cycles of 10 seconds at 95 °C and 30 seconds at 55 °C. Differences between groups were calculated by the 2^−ΔΔCt^ method (34) with *Gapdh* as reference gene. The sequences of the primers used are listed in **Table 1**.

**Table 1:** Primers used for qPCR.

Gene	Forward 5’ – 3’	Reverse 5’ – 3’
*Gapdh*	TAACATCAAATGGGGTGAGG	GGTTCACACCCATCACAAAC
*P2rx7*	GACCGGCGTTGTAAAAAGGG	GGTCGGGGAGCTTCTTTCTC
*CerS6*	GGAGGACCTCTACCTTGCCT	GGATGTTGAGGGCTATGGCA
*Smpd3*	CAACAGCGGTCTCTTCTTCG	TGCAGGCGATGTACCCAA

### Nanoparticle Tracking Analysis

EV suspensions obtained from urine and cell culture medium were analyzed using a NanoSight NS300 and the NanoSight NTA 3.2 software (Malvern Instruments Ltd, Malvern, UK). Three videos of 30 seconds each were recorded for each sample (camera set = 13; threshold = 3). For the analysis of EVs from cell culture medium, particle counts were normalized to the cell count.

### EVs cryo-electron microscopy

EV suspensions obtained by ultracentrifugation were resuspended in 20 µl EV-free PBS. Gold R2/2 Quantifoil grids were cleaned for 40 seconds using a Cressington 208 coater. The sample was applied on a grid and subsequently vitrified in liquid ethane using a FEI Vitrobot device. A JEOL JEM-2100 system (Jeol Ltd., Tokyo, Japan) equipped with Gatan 914 high tilt cryo holder and a LaB_6_ filament was used for cryogenic imaging at 200 kV. Images were recorded with a Gatan 833 Orius camera (Pleasanton, CA, USA).

### RNA sequencing analysis

RNA samples from wild type and *Pkd1^-/-^* mDCT15 cells were obtained as described in 2.6. Total RNA was used for the preparation of the RNA sequencing libraries using the KAPA RNA HyperPrep Kit with RiboErase (KAPA Biosystems, Wilmington, MA, USA). In short, oligo hybridization and rRNA depletion, rRNA depletion cleanup, DNase digestion, DNase digestion cleanup, and RNA elution were performed according to protocol. Fragmentation and priming were performed at 94 °C for 6 minutes. First strand synthesis, second strand synthesis and A-tailing were performed according to protocol. For the adaptor ligation, a 1.5 mM stock was used (NextFlex DNA barcodes, Bioo Scientific, Austin, Tx, USA). First and second post-ligation cleanup was performed according to protocol. A total of 11 PCR cycles were performed for library amplification. The library amplification cleanup was done using a 0.8 x followed by a 1.0 x bead-based cleanup. Library size was determined using the High Sensitivity DNA bioanalyzer kit, and the library concentration was measured using the dsDNA High Sensitivity Assay (DeNovix, Wilmington, DE, USA). Paired-end sequencing reads of 42 bp were generated using an Illumina NextSeq 500. RNA-sequencing data was deposited in the NCBI GEO database (accession number is in progress).

Low-quality filtering and adapter trimming were performed using Trim Galore! v0.4.5 (Babraham Bioinformatics), a wrapper tool around the tools Cutadapt v1.18 and FastQC v0.11.8 (Babraham Bioinformatics). Reads were mapped to a mouse reference genome (GRCm39.105, Ensembl) with Star v2.7.5a, resulting in BAM files. BAM files were counted (number of reads mapped to a feature, e.g., a gene) with HTSeq [HTSeq-count tool v0.11.0] with default parameters using a complementary gtf file, containing annotation for GRCm39.105 (Ensembl). Counts were normalized using gene length and Transcripts Per Million (TPMs) were produced. MultiQC (quality control) was used to combine results and quality checks of all the samples.

Differential gene expression analysis was carried out with DESeq2 v1.22.0 in R v3.5.3 (25), with internal statistical and normalization method (i.e., correction for multiple testing with Benjamini–Hochberg) using a cutoff value of at least 5 counts per sample per gene.

### Statistical analysis

Analyses were performed with GraphPad Prism 7.0e (GraphPad Software, San Diego, USA) and statistical differences were evaluated with the unpaired Student's t-test. All data are expressed as mean ± SEM. For all data, p < 0.05 was considered statistically significant.

## Results

### EV characterization

**Figure 1:**
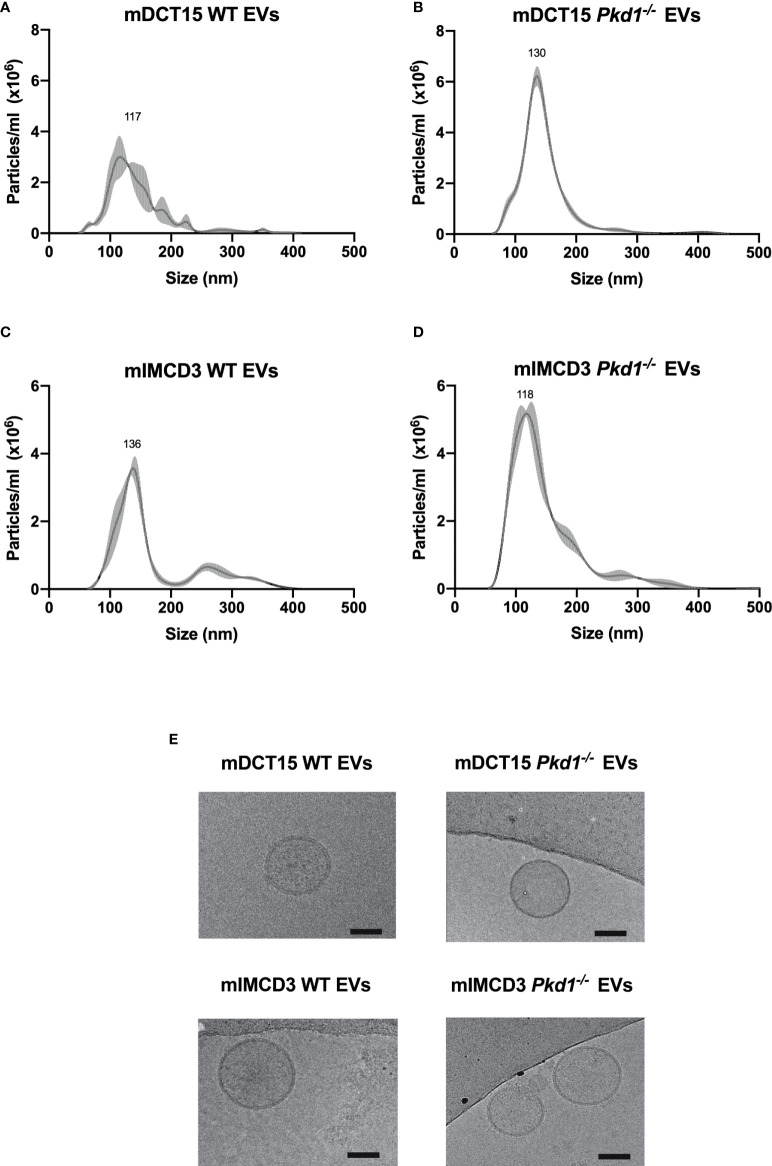
Characterization of EVs obtained from culture medium of mDCT15 and mIMCD3 cells. EVs from culture medium of mDCT15 wild type **(A)** and *Pkd1^-/-^* **(B)** cells and from mIMCD3 wild type **(C)** and *Pkd1^-/-^* **(D)** cells were characterized by NTA and transmission electron microscopy **(E)**. Scale bar: 50 nm.

EVs were isolated from wild type and *Pkd1^-/-^* mDCT15 and mIMCD3 cells by ultracentrifugation. NTA of EVs from wild type and *Pkd1^-/-^* mDCT15 cells showed a peak at 117 nm (mean 117.5 ± 9.7 nm) with a concentration of 2.41 x 10^8^ ± 2.12 x 10^7^ particles/ml (**Figure 1A**) and a peak at 130 nm (mean 130.5 ± 0.4 nm) with a concentration of 3.84 x 10^8^ ± 2.66 x 10^7^ particles/ml (**Figure 1B**), respectively. NTA of EVs from wild type and *Pkd1^-/-^* mIMCD3 cells showed a peak at 136 nm (mean 136.8 ± 3.7 nm) with a concentration of 2.54 x 10^8^ ± 1.57 x 10^7^ particles/ml (**Figure 1C**) and a peak at 118 nm (mean 116.2 ± 7.0 nm) with a concentration of 4.55 x 10^8^ ± 7.18 x 10^6^ particles/ml (**Figure 1D**), respectively.

In addition, the EVs showed the characteristic round morphology with dimensions ranging between 50 and 150 nm, as depicted in the cryogenic transmission electron micrographs (**Figure 1E**).

### Increase in EV count in *Pkd1^-/-^* models

**Figure 2:**
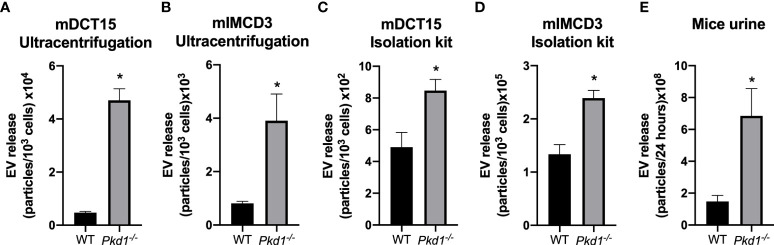
Increase in EV release in *Pkd1^-/-^* models *in vitro* and *in vivo*. The concentration of EVs was analyzed by NTA in preparations obtained by ultracentrifugation from mDCT15 **(A)** and mIMCD3 cells **(B)** and by precipitation with the Total Exosome Isolation Reagent (from cell culture medium) from mDCT15 **(C)** and mIMCD3 cells **(D)**. The particle concentration was normalized to the total cell count. Data are shown as mean ± SEM (n = 3). Urinary EVs in samples from *Pkd1^-/-^* and wild type (WT) mice were isolated with the Total Exosome Isolation Reagent (from urine) and analyzed by NTA. Data are shown as mean ± SEM (n = 8 for WT mice and n = 5 for *Pkd1^-/-^* mice) **(E)**. * indicates statistical difference from WT (p < 0.05).

The loss of *Pkd1* expression was associated with an increase in the EV release in both mDCT15 (**Figure 2A**) and mIMCD3 cells (**Figure 2B**), as determined by NTA of EV suspensions obtained through ultracentrifugation. This result was confirmed for EV suspensions from mDCT15 (**Figure 2C**) and mIMCD3 cells (**Figure 2D**) obtained using the total exosome isolation kit (from cell culture), which is based on EV precipitation. In the same line, urine from *iKsp*-*Pkd1^-/-^* mice exhibited a significant increase in urinary EVs compared to the wild type littermates (**Figure 2E**).

### Regulation of EV release by extracellular ATP

**Figure 3:**
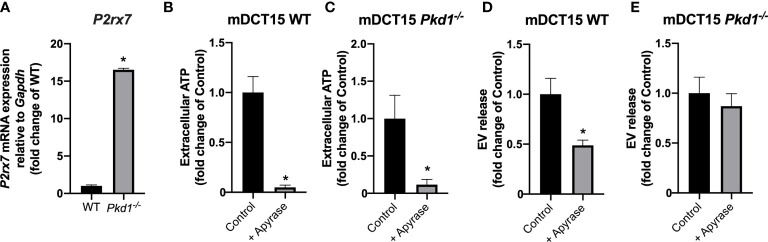
Role of the extracellular ATP and purinergic signaling in EV release in wild type and *Pkd1^-/-^* mDCT15 cells. Expression of *P2rx7* at the mRNA level was analyzed in wild type and *Pkd1^-/-^* mDCT15 cells and normalized to the expression of *Gapdh*, used as housekeeping gene **(A)**. Data (mean ± SEM) are expressed as fold change of the gene expression in wild type (WT) cells. * indicates statistical difference from WT (p < 0.05, n = 3). Extracellular ATP levels were analyzed in WT **(B)** and *Pkd1^-/-^* mDCT15 cells **(C)** exposed to vehicle (control) or apyrase (6 U/ml, 24 hours). Data (mean ± SEM) are expressed as fold change of extracellular ATP levels in control cells. EV release was quantified in suspensions obtained from wild type **(D)** and *Pkd1^-^/^-^* mDCT15 cells **(E)** treated with vehicle (control) or with 6 U/ml apyrase by NTA. Data are shown as mean ± SEM. * indicates statistical difference from control (p < 0.05, n = 4).

The molecular mechanisms underlying the increase in EV release in *Pkd1^-/-^* models was investigated in mDCT15 cells. The loss of *Pkd1* was associated with a significant increase in the gene expression of the purinergic receptor *P2rx7* in mDCT15 cells (**Figure 3A**). To further assess the potential role of purinergic signaling in the increase in the EV release previously observed, we used apyrase to deplete the culture medium from extracellular ATP. In fact, treatment with apyrase led to a significant decrease of extracellular ATP levels in both wild type and *Pkd1^-/-^* cells (**Figures 3B, C**). Furthermore, apyrase treatment resulted in a significant decrease in the EV release in the wild type mDCT15 cells (**Figure 3D**), while EV release in mDCT15 *Pkd1^-/-^* cells was not modified by apyrase (**Figure 3E**). These observations evidence the regulation of EV release in response to changing extracellular ATP levels in wild type mDCT15 cells. Conversely, changing levels of extracellular ATP fail to regulate EV release in *Pkd1^-/-^* mDCT15 cells.

### Role of the sphingolipid biosynthesis on EVs release in mDCT15 *Pkd1^-/-^* cells

**Figure 4:**
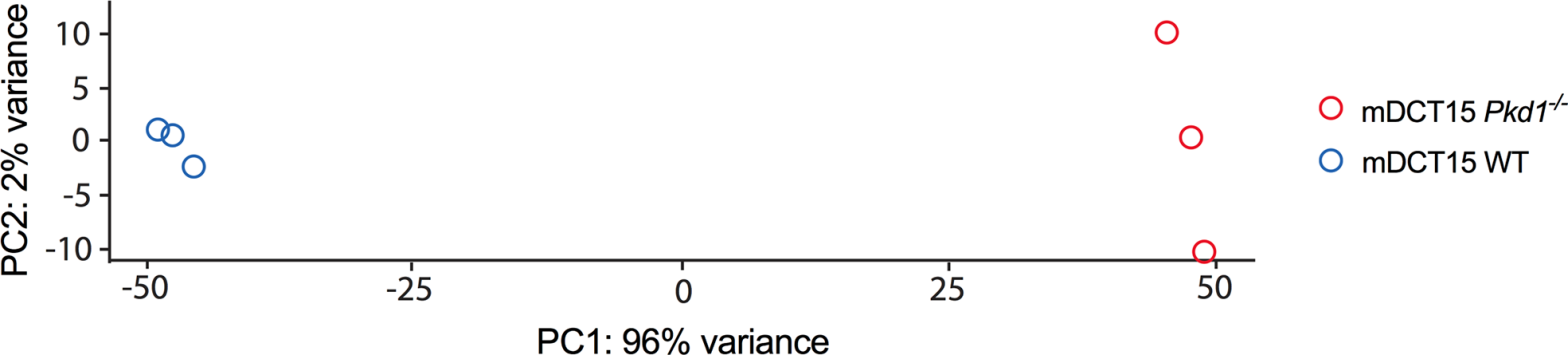
Principal component analysis of the RNAseq data transcripts of wild type and *Pkd1^-/-^* mDCT15 cells. The plot depicts a clear separation between the *Pkd1^-/-^* mDCT15 cell samples (red circles) and the wild type mDCT15 cell samples (blue circles) (n = 3/group).

**Figure 5:**
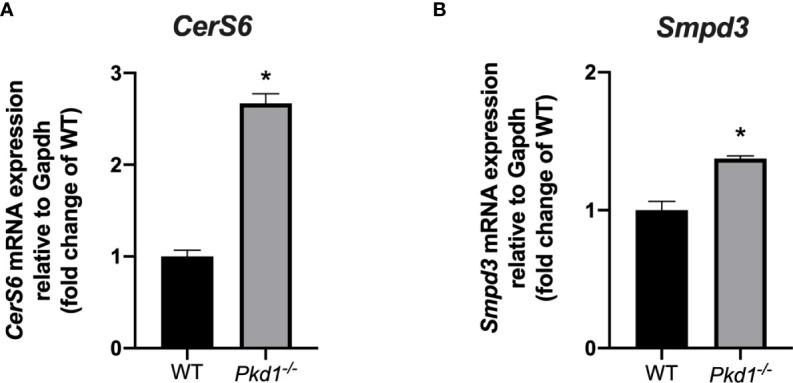
Effect of *Pkd1* knockout on the expression of ceramide biosynthesis enzymes. The expression of the *CerS6* **(A)** and *Smpd3* **(B)** in wild type (WT) and *Pkd1^-/-^* mDCT15 cells was analyzed by qPCR and normalized to the expression of *Gapdh*, used as housekeeping gene. Data (mean ± SEM) are expressed as fold change of the expression in the wild type cells. * indicates statistical difference from WT (p < 0.05, n = 3).

To further elucidate the molecular players responsible for the increased EV release in *Pkd1* deficient cells, we performed RNA sequencing analysis of wild type and *Pkd1^-/-^* mDCT15 cells. Principal component analysis showed a clear separation between wild type and *Pkd1^-/-^* mDCT15 cells (Principal Component 1 = 96%, Principal Component 2 = 2%) (**Figure 4**). RNA sequencing data exhibited a total of 1,163 differentially expressed genes (DEGS) with fold change > 2 and p < 0.05 (**Supplementary Material 1**). Subsequent analysis of the latter revealed several significantly enriched KEGG pathways (adjusted p-value < 0.01) (**Table 2**) that included the term “Sphingolipid signaling pathway”. To further investigate the potential role of sphingolipid biosynthesis in EV release in wild type and *Pkd1^-/-^* mDCT15 cells, we quantified the gene expression level of *CerS6*, which encodes the enzyme ceramide synthase 6 and *Smpd3*, encoding the neutral sphingomyelinase 3. We observed a significant increase in the expression of both *CerS6* (+ 167 %) (**Figure 5A**) and *Smpd3* (+ 38%) (**Figure 5B**) in *Pkd1^-/-^* mDCT15 cells compared to wild type cells. Noteworthy, an upregulation of both *CerS6* and *Smpd3* was also observed in the RNA sequencing data (**Supplementary Material 1**). Other genes, encoding ceramide synthases, that were upregulated in *Pkd1^-/-^* mDCT15 cells included *CerS2* and *CerS5*. Anyway, accordingly to the RNA sequencing results, CerS6 represented the most differentiated gene in that group.

**Table 2:** KEGG terms of genes differentially expressed in wild type and *Pkd1^-/-^* mDCT15 cells.

KEGG pathway	Adjusted p-value (q value < 0.01)
Proteoglycans in cancer	2.83E-09
Ubiquitin mediated proteolysis	2.93E-08
Hepatocellular carcinoma	1.32E-06
Colorectal cancer	7.71E-06
Hepatitis B	2.98E-05
Renal cell carcinoma	3.26E-05
Proteasome	3.35E-05
Cell cycle	3.36E-05
Axon guidance	3.37E-05
Endometrial cancer	3.41E-05
Signaling pathways regulating pluripotency of stem cells	4.34E-05
Autophagy	5.05E-05
Pathways of neurodegeneration	6.97E-05
mTOR signaling pathway	6.97E-05
Chronic myeloid leukemia	6.97E-05
MAPK signaling pathway	7.06E-05
Pathways in cancer	7.06E-05
Alzheimer disease	7.06E-05
Protein processing in endoplasmic reticulum	7.69E-05
Small cell lung cancer	9.75E-05
Neurotrophin signaling pathway	1.34E-04
Salmonella infection	1.70E-04
Focal adhesion	2.39E-04
Regulation of actin cytoskeleton	2.64E-04
Cellular senescence	3.26E-04
ErbB signaling pathway	3.87E-04
Yersinia infection	4.95E-04
Human T-cell leukemia virus 1 infection	5.17E-04
Amyotrophic lateral sclerosis	6.89E-04
Pancreatic cancer	6.89E-04
Pathogenic Escherichia coli infection	6.89E-04
Endocytosis	6.89E-04
Human papillomavirus infection	8.12E-04
TNF signaling pathway	8.13E-04
Human cytomegalovirus infection	9.50E-04
Prostate cancer	9.67E-04
Glioma	9,67E-04
Inositol phosphate metabolism	1.01E-03
Acute myeloid leukemia	1.21E-03
Gastric cancer	1.46E-03
Sphingolipid signaling pathway	1.48E-03
Non-small cell lung cancer	1.49E-03
Epithelial cell signaling in Helicobacter pylori infection	1.57E-03
Mitophagy	1.65E-03
C-type lectin receptor signaling pathway	1.84E-03
HIF-1 signaling pathway	2.07E-03
Hippo signaling pathway	2.20E-03
Apoptosis	2.29E-03
Breast cancer	2.42E-03
FoxO signaling pathway	2.67E-03
Oocyte meiosis	2.87E-03
VEGF signaling pathway	3.06E-03
Phosphatidylinositol signaling system	3.08E-03
Ribosome	3.20E-03
AGE-RAGE signaling pathway in diabetic complications	3.70E-03
Kaposi sarcoma-associated herpesvirus infection	4.15E-03
Hepatitis C	4.21E-03
Thyroid cancer	5.29E-03
Basal cell carcinoma	5.62E-03
Human immunodeficiency virus 1 infection	5.69E-03
Thyroid hormone signaling pathway	6.45E-03
Bacterial invasion of epithelial cells	6.45E-03
RNA transport	6.45E-03
Spinocerebellar ataxia	7.10E-03
PD-L1 expression and PD-1 checkpoint pathway in cancer	7.28E-03
Shigellosis	7.28E-03
Parkinson disease	7.63E-03
Epstein-Barr virus infection	7.63E-03
Adherens junction	7.63E-03
AMPK signaling pathway	7.96E-03
Insulin signaling pathway	8.33E-03
Wnt signaling pathway	8.33E-03
Fatty acid degradation	9.91E-03
Melanoma	9.91E-03

Differentially expressed genes with p < 0.05 and foldchange > 2 were analyzed.

## Discussion

In the current study, we describe an increase in the EV release by *Pkd1^-/-^* mIMCD3 and mDCT15 cells compared to wild type cells. Moreover, we observed an increase in the urinary EV concentration in *Pkd1^-/-^* mice. EVs have been proposed as mediators of cell-cell communication, which contribute to the progression of ADPKD. In particular, urinary EVs from ADPKD patients stimulated cell proliferation and cyst development in an *in vitro* 3D cyst model. In addition, an increase in the EV concentration in the urine of ADPKD patients has been reported (28). An increase in EV release in *Pkd1^-/-^* cells respect to *Pkd2^-/-^* cells was also described in mIMCD3 cells (27). Altogether, our findings confirm these previous findings from other groups (27, 28) which relate EVs with ADPKD pathogenesis. Interestingly, we highlight for the first time the contribution of the DCT to the increased EV release under *Pkd1* deficiency. Recently, a single cell RNA-sequencing study confirmed the participation of all segments of the nephron in the cystogenesis (35), whereby the signaling pathways involved were demonstrated to be different between cell types. Noteworthy, in this study, the DCT was partially analyzed as a cluster with the loop of Henle (35). Considering the available evidence on the role of EVs on cyst progression, our findings demonstrating the increased EV release by *Pkd1^-/-^* DCT cells support a role of this segment in ADPKD pathogenesis.

To further elucidate the molecular mechanisms underlying the increase in the EV release in *Pkd1^-/-^* mDCT15 cells, we assessed the role of purinergic signaling, a pathway already demonstrated to modulate EV release in different cell types, albeit not in the kidney. In general, an increase in extracellular ATP and activation of the receptor P2X7 results in an increase in EV release (23–25). Our data depict a notable increase in *P2rx7* expression in *Pkd1^-/-^* mDCT15 cells compared to wild type cells, indicating a stimulation of purinergic signaling by *Pkd1* deficiency. Moreover, EV release in *Pkd1^-/-^* cells was not inhibited by the ectonucleotidase apyrase (i.e. reduced extracellular ATP levels), contrarily to what has been observed in wild type mDCT15 cells. These observations point to a sustained activation of purinergic signaling and EV release due to upregulated *P2rx7* expression in *Pkd1^-/-^* mDCT15 cells. Thus, the lack of *Pkd1* expression might lead to an exacerbation of the extracellular ATP/P2X7 pathway in *Pkd1^-/-^* mDCT15 cells, which then become less responsive to changes in extracellular ATP levels and therefore, exhibit no changes in their EV release. The association between *P2rx7* and cystogenesis has also been described by other authors. For instance, a study in a zebrafish model showed that activation and inhibition of the receptor resulted in stimulation and inhibition of cystogenesis, respectively (17).

Interestingly, previous publications demonstrated an increase in ceramide levels due to *de novo* synthesis upon activation of *P2rx7* in thymocytes (36) and macrophages (37). No studies have been performed in renal cells so far. Ceramides constitute an important component of EVs and ceramide levels correlate with EV release (38). Our RNA-sequencing analysis indicated a significant upregulation of genes involved in ceramide biosynthesis (i.e. *CerS6* and *Smpd3*) in *Pkd1^-/-^* mDCT15 cells respect to wild type cells. In this regard, ceramides may constitute the missing link between increased purinergic signaling, increased EV release and cystogenesis in ADPKD. Indeed, further studies would still be necessary to further confirm the link between ceramide production and the expression of *Pkd1^-/-^* in relation to EV release, including analysis of the phenotype in an overexpression rescue model.

**Figure 6:**
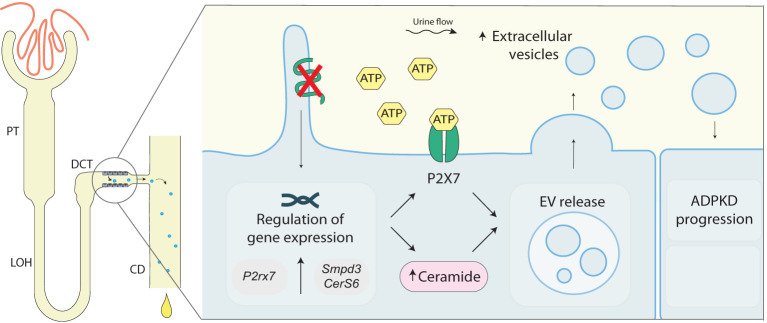
Proposed role of polycystin-1 deficiency in EV release in the distal convoluted tubule. Loss of *Pkd1^-/-^* in mDCT15 cells is associated with an increase in the EV release and an increase in the expression of *P2xr7*, S*mpd3* and *CerS6*, genes encoding for proteins involved in ATP signaling and ceramide synthesis, respectively. These findings suggest a link between the loss of *Pkd1* expression, ceramide biosynthesis and EV release. The latter can stimulate cysts development in the surrounding tissue contributing to ADPKD disease progression. ADPKD, Autosomal dominant polycystic kidney disease; CD, collecting duct; *CerS6*, Ceramide Synthase 6; DCT, distal convoluted tubule; EV, extracellular vesicles; LOH, Loop of Henle; PC1, polycystin 1; PT, proximal tubule; *P2rx7*, Purinergic Receptor P2X 7 (gene); P2X7, Purinergic Receptor P2X 7 (protein); *Smpd3*, Sphingomyelin Phosphodiesterase 3.

Although several factors involved in ADPKD progression are not yet elucidated, a model mimicking a snowball-effect has been proposed. In this model, cysts formed at an early stage of the disease stimulate further cyst formation in the surrounding tissue and, thus, lead to an increasing cyst formation rate which leads to disease progression (39). Hereby, exacerbated purinergic signaling could lead to the upregulation of ceramide biosynthesis. Increased ceramide levels can lead, in turn, to increased EV release, which can stimulate cyst development in the surrounding tissue. A proposed mechanism has been depicted in **Figure 6**. In conclusion, our data points to the involvement of the DCT in cyst progression and development and postulates purinergic receptor activation together with increased ceramide biosynthesis as one of the underlying mechanisms.

## Data availability statement

The data presented in the study are deposited in the Gene Expression Omibus repository, accession number GSE209927.

## Author contributions

VC participated in the design of the study, performed the experimental work and in writing of the manuscript. LR, NS and CK provided experimental and theoretical support. EV provided the material. VS participated in the design of the study and the experimental work. JPR was involved in the design of the study, the interpretation of the data, the experimental work and in the manuscript writing. JW and JH participated in the study design, the interpretation of the data and the editing of the manuscript. All authors contributed to the article and approved the submitted version.

## Funding

The research has been funded by a PhD research grant from the Radboud Institute for Molecular Life Sciences within the Radboud university medical center. JR is funded by a grant from the German Research Foundation (DFG, Grant RI2673/2-1, Project number: 509856975) and by the European Union’s Horizon 2020 research and innovation programme under the Marie Sklodowska-Curie grant agreement No 101028473. NS and LR are supported by the European Research Council (ERC) Advanced Investigator grant (H2020-ERC-2017-ADV-788982- COLMIN) which was granted to NS.

## Acknowledgments

The authors would like to thank Dr. Robert Hoover (Emory University, Atlanta, USA) for kindly providing mDCT15 cells and Dr. Dorien Peters (LUMC, Leiden, the Netherlands) for kindly providing wild type and *Pkd1^-/-^* mIMCD3 cells as well as Dr. Joost Martens (Radboudumc, Nijmegen, the Netherlands) for the preparation of the libraries.

## Conflict of interest

The authors declare that the research was conducted in the absence of any commercial or financial relationships that could be construed as a potential conflict of interest.

## Publisher’s note

All claims expressed in this article are solely those of the authors and do not necessarily represent those of their affiliated organizations, or those of the publisher, the editors and the reviewers. Any product that may be evaluated in this article, or claim that may be made by its manufacturer, is not guaranteed or endorsed by the publisher.
